# {2,2′-[(Benzyl­aza­nedi­yl)dimethylene]diphenolato}(methano­lato)boron

**DOI:** 10.1107/S1600536811016990

**Published:** 2011-05-11

**Authors:** Yanxia Geng, Wangsuo Wu

**Affiliations:** a Radiochemistry Laboratory, School of Nuclear Science and Technology, Lanzhou University, Lanzhou 730000, People’s Republic of China

## Abstract

The title compound, C_22_H_22_BNO_3_, was unintentionally obtained from salicyl­aldehyde benzyl­amine and sodium borohydride. The B—O bond lengths lie in the range 1.425 (2)–1.463 (2) Å, and B—N = 1.641 (2) Å. In the crystal, weak inter­molecular C—H⋯O hydrogen bonds link the mol­ecules into chains in the [010] direction.

## Related literature

For the crystal structure of a related compound, see: Muller & Burgi (1987 ▶).
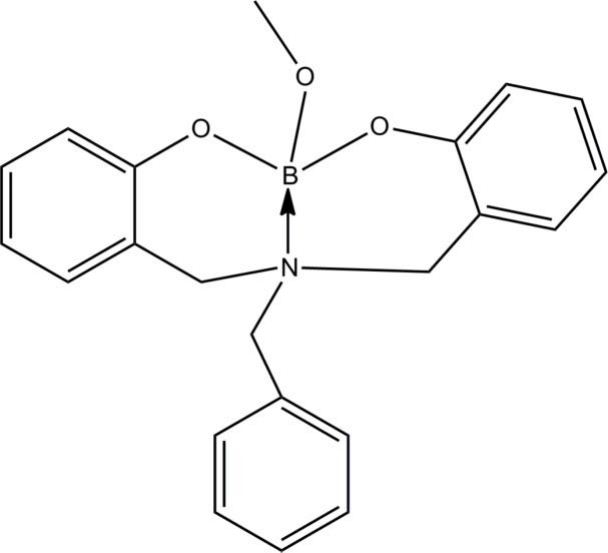


## Experimental

### 

#### Crystal data

C_22_H_22_BNO_3_
*M* *_r_* = 359.22
Monoclinic, 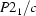

*a* = 12.5041 (14) Å
*b* = 10.6029 (12) Å
*c* = 17.1420 (14) Å
β = 124.054 (5)°
*V* = 1882.9 (3) Å^3^
*Z* = 4
Mo *K*α radiation
μ = 0.08 mm^−1^
*T* = 273 K
0.31 × 0.26 × 0.22 mm

#### Data collection

Bruker SMART APEX diffractometer
Absorption correction: multi-scan (*SADABS*; Bruker, 2005 ▶) *T*_min_ = 0.975, *T*_max_ = 0.982
11689 measured reflections
4540 independent reflections
2558 reflections with *I* > 2σ(*I*)
*R*_int_ = 0.154

#### Refinement

*R*[*F* ^2^ > 2σ(*F* ^2^)] = 0.058
*wR*(*F* ^2^) = 0.161
*S* = 1.01
4540 reflections
245 parameters
H-atom parameters constrained
Δρ_max_ = 0.23 e Å^−3^
Δρ_min_ = −0.24 e Å^−3^

### 

Data collection: *SMART* (Bruker, 2005 ▶); cell refinement: *SAINT* (Bruker, 2005 ▶); data reduction: *SAINT*; program(s) used to solve structure: *SHELXS97* (Sheldrick, 2008 ▶); program(s) used to refine structure: *SHELXL97* (Sheldrick, 2008 ▶); molecular graphics: *XP* in *SHELXTL* (Sheldrick, 2008 ▶); software used to prepare material for publication: *SHELXL97*.

## Supplementary Material

Crystal structure: contains datablocks I, global. DOI: 10.1107/S1600536811016990/cv5081sup1.cif

Structure factors: contains datablocks I. DOI: 10.1107/S1600536811016990/cv5081Isup2.hkl

Additional supplementary materials:  crystallographic information; 3D view; checkCIF report

## Figures and Tables

**Table 1:** Hydrogen-bond geometry (Å, °)

*D*—H⋯*A*	*D*—H	H⋯*A*	*D*⋯*A*	*D*—H⋯*A*
C22—H22⋯O5^i^	0.93	2.54	3.399 (2)	153

Symmetry code: (i) 
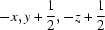
.

**Fig. 1.**
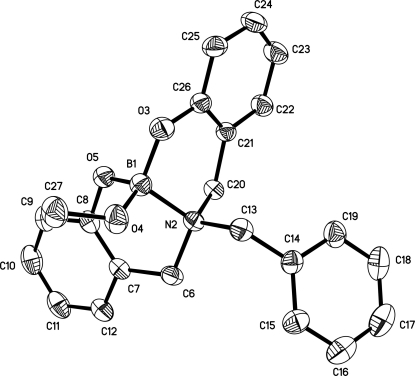
The molecular structure of title compound showing the atomic numbering and 30% probability displacement ellipsoids. H-atoms omitted for clarity.

## supplementary crystallographic information

### Comment

The title compound, (I), has been unintentionally obtained during the attempts to synthesize the metal complexes with phenolamine derivatives.

In (I), all bond lengths and angles are normal and comparable with those observed in 2,2',2''-nitrilotriphenyl borate(III) (Muller & Burgi, 1987). The B1—N2 bond of 1.642 (2) Å indicates strong contact due to the electric charge action. Weak intermolecular C—H···O hydrogen bonds (Table 1) link the molecules into chains in [010].

### Experimental

The salicylaldehyde (4.4 ml, 0.04 mol) was disolved in 25 ml anhydrous methanol, then benzylamine 4.4 ml(0. 04 mol) disolved in 15 ml anhydrous methnol was added to the former by drops.After reacted under ultrasonography for 40 min, sodium borohydride (1.5 g, 0.04 mol) were added in batch. After reacted under ultrasonography for another 20 min, 1, 4-dibromo-butane(2.4 ml, 0.02 mol) were added, After stirring of 24 h at room temperature. The precipitate was filtered off and dried. The single-crystal suitable for X-ray diffraction analysis was obtained by recrystallization from methanol.

The yield is 75% and elemental analysis: calc. for C_22_H_22_BNO_3_: C 73.55, H 6.17, N 3.90; found: C 72.81, H 6.49, N 3.53. The elemental analyses were performed with PERKIN ELMER MODEL 2400 SERIES II.

### Refinement

H atoms were geometrically positioned (C—H 0.93-0.97 Å) and refined as riding, with *U*_iso_(H) = 1.2-1.5 *U*_eq_(C).

### Crystal data

**Table:**

C_22_H_22_BNO_3_	*F*(000) = 760
*M**_r_* = 359.22	*D*_x_ = 1.267 Mg m^−^^3^
Monoclinic, *P*2_1_/*c*	Mo *K*α radiation, λ = 0.71073 Å
*a* = 12.5041 (14) Å	Cell parameters from 2910 reflections
*b* = 10.6029 (12) Å	θ = 2.4–23.2°
*c* = 17.1420 (14) Å	µ = 0.08 mm^−^^1^
β = 124.054 (5)°	*T* = 273 K
*V* = 1882.9 (3) Å^3^	Block, colourless
*Z* = 4	0.31 × 0.26 × 0.22 mm

### Data collection

**Table:**

Bruker SMART APEX diffractometer	4540 independent reflections
Radiation source: fine-focus sealed tube	2558 reflections with *I* > 2σ(*I*)
graphite	*R*_int_ = 0.154
φ and ω scans	θ_max_ = 28.4°, θ_min_ = 2.0°
Absorption correction: multi-scan (*SADABS*; Bruker, 2005)	*h* = −16→16
*T*_min_ = 0.975, *T*_max_ = 0.982	*k* = −13→14
11689 measured reflections	*l* = −19→22

### Refinement

**Table:**

Refinement on *F*^2^	Primary atom site location: structure-invariant direct methods
Least-squares matrix: full	Secondary atom site location: difference Fourier map
*R*[*F*^2^ > 2σ(*F*^2^)] = 0.058	Hydrogen site location: inferred from neighbouring sites
*wR*(*F*^2^) = 0.161	H-atom parameters constrained
*S* = 1.01	*w* = 1/[σ^2^(*F*_o_^2^) + (0.04*P*)^2^] where *P* = (*F*_o_^2^ + 2*F*_c_^2^)/3
4540 reflections	(Δ/σ)_max_ = 0.003
245 parameters	Δρ_max_ = 0.23 e Å^−^^3^
0 restraints	Δρ_min_ = −0.24 e Å^−^^3^

### Special details

**Table:**

Geometry. All e.s.d.'s (except the e.s.d. in the dihedral angle between two l.s. planes) are estimated using the full covariance matrix. The cell e.s.d.'s are taken into account individually in the estimation of e.s.d.'s in distances, angles and torsion angles; correlations between e.s.d.'s in cell parameters are only used when they are defined by crystal symmetry. An approximate (isotropic) treatment of cell e.s.d.'s is used for estimating e.s.d.'s involving l.s. planes.
Refinement. Refinement of *F*^2^ against ALL reflections. The weighted *R*-factor *wR* and goodness of fit *S* are based on *F*^2^, conventional *R*-factors *R* are based on *F*, with *F* set to zero for negative *F*^2^. The threshold expression of *F*^2^ > σ(*F*^2^) is used only for calculating *R*-factors(gt) *etc*. and is not relevant to the choice of reflections for refinement. *R*-factors based on *F*^2^ are statistically about twice as large as those based on *F*, and *R*- factors based on ALL data will be even larger.

### Fractional atomic coordinates and isotropic or equivalent isotropic displacement parameters (Å^2^)

**Table:**

	*x*	*y*	*z*	*U*_iso_*/*U*_eq_	
B1	0.2232 (2)	0.32181 (19)	0.40625 (14)	0.0467 (5)	
N2	0.20528 (12)	0.47562 (12)	0.39796 (8)	0.0405 (3)	
O3	0.11650 (13)	0.26732 (11)	0.40506 (9)	0.0542 (3)	
O4	0.34523 (13)	0.29948 (12)	0.49191 (9)	0.0613 (4)	
O5	0.21491 (12)	0.28152 (10)	0.32141 (8)	0.0498 (3)	
C6	0.31610 (16)	0.53161 (17)	0.39892 (12)	0.0487 (4)	
H6A	0.3922	0.5319	0.4630	0.058*	
H6B	0.2959	0.6184	0.3776	0.058*	
C7	0.34535 (16)	0.45969 (17)	0.33689 (12)	0.0473 (4)	
C8	0.29429 (17)	0.34032 (18)	0.30145 (12)	0.0488 (4)	
C9	0.3203 (2)	0.27914 (19)	0.24196 (14)	0.0623 (5)	
H9	0.2839	0.2008	0.2167	0.075*	
C10	0.4007 (2)	0.3356 (2)	0.22079 (16)	0.0734 (6)	
H10	0.4182	0.2947	0.1811	0.088*	
C11	0.4551 (2)	0.4515 (2)	0.25773 (15)	0.0704 (6)	
H11	0.5105	0.4880	0.2442	0.085*	
C12	0.42708 (19)	0.51334 (19)	0.31478 (14)	0.0574 (5)	
H12	0.4632	0.5922	0.3390	0.069*	
C13	0.20248 (18)	0.52595 (15)	0.47992 (11)	0.0475 (4)	
H13A	0.1326	0.4849	0.4792	0.057*	
H13B	0.2825	0.5025	0.5382	0.057*	
C14	0.18550 (18)	0.66690 (17)	0.48009 (11)	0.0481 (4)	
C15	0.2926 (2)	0.74533 (18)	0.52878 (14)	0.0606 (5)	
H15	0.3750	0.7106	0.5607	0.073*	
C16	0.2782 (3)	0.8744 (2)	0.53036 (17)	0.0793 (7)	
H16	0.3508	0.9256	0.5632	0.095*	
C17	0.1563 (3)	0.9279 (2)	0.48346 (17)	0.0815 (7)	
H17	0.1467	1.0148	0.4836	0.098*	
C18	0.0494 (3)	0.8508 (2)	0.43666 (15)	0.0761 (7)	
H18	−0.0328	0.8858	0.4059	0.091*	
C19	0.0638 (2)	0.7210 (2)	0.43516 (14)	0.0626 (6)	
H19	−0.0090	0.6698	0.4037	0.075*	
C20	0.08093 (15)	0.50238 (15)	0.30599 (11)	0.0416 (4)	
H20A	0.0898	0.4816	0.2548	0.050*	
H20B	0.0613	0.5916	0.3019	0.050*	
C21	−0.02795 (16)	0.42715 (15)	0.29604 (11)	0.0429 (4)	
C22	−0.15376 (18)	0.47161 (18)	0.23826 (13)	0.0558 (5)	
H22	−0.1697	0.5459	0.2046	0.067*	
C23	−0.2553 (2)	0.4070 (2)	0.23014 (16)	0.0703 (6)	
H23	−0.3390	0.4377	0.1913	0.084*	
C24	−0.2323 (2)	0.2971 (2)	0.27968 (17)	0.0719 (7)	
H24	−0.3006	0.2543	0.2750	0.086*	
C25	−0.1073 (2)	0.24931 (18)	0.33692 (15)	0.0598 (5)	
H25	−0.0925	0.1742	0.3696	0.072*	
C26	−0.00545 (18)	0.31408 (16)	0.34496 (12)	0.0457 (4)	
C27	0.4118 (2)	0.18849 (19)	0.50173 (16)	0.0716 (6)	
H27A	0.3651	0.1176	0.5031	0.107*	
H27B	0.4961	0.1915	0.5593	0.107*	
H27C	0.4199	0.1800	0.4495	0.107*	

### Atomic displacement parameters (Å^2^)

**Table:**

	*U*^11^	*U*^22^	*U*^33^	*U*^12^	*U*^13^	*U*^23^
B1	0.0461 (12)	0.0474 (11)	0.0464 (11)	0.0016 (9)	0.0258 (10)	0.0020 (8)
N2	0.0369 (8)	0.0476 (8)	0.0388 (7)	−0.0015 (6)	0.0222 (7)	0.0012 (6)
O3	0.0582 (8)	0.0479 (7)	0.0628 (8)	−0.0006 (6)	0.0378 (7)	0.0096 (6)
O4	0.0543 (8)	0.0651 (8)	0.0502 (8)	0.0158 (7)	0.0206 (7)	0.0049 (6)
O5	0.0488 (7)	0.0512 (7)	0.0520 (7)	−0.0032 (6)	0.0299 (7)	−0.0048 (5)
C6	0.0384 (9)	0.0544 (10)	0.0531 (10)	−0.0058 (8)	0.0255 (9)	−0.0022 (8)
C7	0.0369 (9)	0.0574 (11)	0.0482 (10)	0.0032 (8)	0.0242 (8)	0.0059 (8)
C8	0.0426 (10)	0.0573 (11)	0.0467 (10)	0.0070 (9)	0.0250 (9)	0.0051 (8)
C9	0.0661 (13)	0.0670 (13)	0.0611 (13)	0.0116 (11)	0.0401 (12)	0.0007 (10)
C10	0.0798 (16)	0.0905 (16)	0.0720 (14)	0.0220 (14)	0.0561 (14)	0.0102 (12)
C11	0.0619 (13)	0.0920 (16)	0.0760 (14)	0.0161 (13)	0.0500 (12)	0.0216 (13)
C12	0.0461 (10)	0.0665 (12)	0.0632 (12)	0.0048 (10)	0.0328 (10)	0.0104 (10)
C13	0.0478 (10)	0.0552 (10)	0.0413 (9)	−0.0022 (8)	0.0260 (8)	−0.0008 (7)
C14	0.0531 (11)	0.0531 (10)	0.0394 (9)	0.0005 (9)	0.0267 (9)	−0.0028 (8)
C15	0.0637 (13)	0.0605 (12)	0.0525 (12)	−0.0071 (11)	0.0293 (11)	−0.0051 (9)
C16	0.1020 (19)	0.0619 (14)	0.0735 (15)	−0.0152 (14)	0.0489 (15)	−0.0104 (11)
C17	0.122 (2)	0.0552 (12)	0.0719 (15)	0.0098 (16)	0.0575 (16)	0.0010 (11)
C18	0.0910 (17)	0.0770 (15)	0.0596 (13)	0.0318 (15)	0.0417 (13)	0.0044 (11)
C19	0.0575 (12)	0.0742 (14)	0.0526 (12)	0.0079 (11)	0.0287 (11)	−0.0077 (10)
C20	0.0381 (9)	0.0464 (9)	0.0410 (9)	0.0011 (7)	0.0226 (8)	0.0029 (7)
C21	0.0409 (9)	0.0472 (9)	0.0427 (9)	−0.0056 (8)	0.0247 (8)	−0.0069 (7)
C22	0.0429 (10)	0.0646 (12)	0.0541 (11)	−0.0019 (9)	0.0236 (9)	−0.0065 (9)
C23	0.0416 (11)	0.0870 (16)	0.0757 (14)	−0.0103 (11)	0.0288 (11)	−0.0199 (12)
C24	0.0570 (14)	0.0882 (17)	0.0850 (16)	−0.0296 (12)	0.0487 (13)	−0.0329 (13)
C25	0.0733 (14)	0.0550 (11)	0.0709 (14)	−0.0205 (11)	0.0524 (13)	−0.0153 (9)
C26	0.0474 (10)	0.0471 (10)	0.0495 (10)	−0.0072 (8)	0.0314 (9)	−0.0081 (8)
C27	0.0639 (14)	0.0677 (13)	0.0718 (14)	0.0189 (11)	0.0309 (13)	0.0179 (10)

### Geometric parameters (Å, °)

**Table:**

B1—O4	1.425 (2)	C14—C15	1.389 (3)
B1—O3	1.443 (2)	C15—C16	1.382 (3)
B1—O5	1.463 (2)	C15—H15	0.9300
B1—N2	1.641 (2)	C16—C17	1.385 (3)
N2—C20	1.4962 (19)	C16—H16	0.9300
N2—C6	1.499 (2)	C17—C18	1.377 (3)
N2—C13	1.521 (2)	C17—H17	0.9300
O3—C26	1.369 (2)	C18—C19	1.390 (3)
O4—C27	1.396 (2)	C18—H18	0.9300
O5—C8	1.368 (2)	C19—H19	0.9300
C6—C7	1.510 (2)	C20—C21	1.503 (2)
C6—H6A	0.9700	C20—H20A	0.9700
C6—H6B	0.9700	C20—H20B	0.9700
C7—C12	1.395 (2)	C21—C22	1.390 (3)
C7—C8	1.394 (3)	C21—C26	1.398 (2)
C8—C9	1.392 (3)	C22—C23	1.379 (3)
C9—C10	1.381 (3)	C22—H22	0.9300
C9—H9	0.9300	C23—C24	1.374 (3)
C10—C11	1.376 (3)	C23—H23	0.9300
C10—H10	0.9300	C24—C25	1.394 (3)
C11—C12	1.375 (3)	C24—H24	0.9300
C11—H11	0.9300	C25—C26	1.385 (3)
C12—H12	0.9300	C25—H25	0.9300
C13—C14	1.510 (2)	C27—H27A	0.9600
C13—H13A	0.9700	C27—H27B	0.9600
C13—H13B	0.9700	C27—H27C	0.9600
C14—C19	1.388 (3)		
			
O4—B1—O3	113.34 (15)	C15—C14—C13	120.28 (17)
O4—B1—O5	114.47 (16)	C16—C15—C14	120.7 (2)
O3—B1—O5	108.60 (15)	C16—C15—H15	119.6
O4—B1—N2	105.64 (14)	C14—C15—H15	119.6
O3—B1—N2	108.45 (14)	C15—C16—C17	120.5 (2)
O5—B1—N2	105.89 (13)	C15—C16—H16	119.7
C20—N2—C6	110.19 (12)	C17—C16—H16	119.7
C20—N2—C13	111.03 (13)	C18—C17—C16	119.2 (2)
C6—N2—C13	110.50 (13)	C18—C17—H17	120.4
C20—N2—B1	106.88 (12)	C16—C17—H17	120.4
C6—N2—B1	108.19 (13)	C17—C18—C19	120.3 (2)
C13—N2—B1	109.94 (12)	C17—C18—H18	119.8
C26—O3—B1	119.80 (13)	C19—C18—H18	119.8
C27—O4—B1	119.10 (15)	C14—C19—C18	120.8 (2)
C8—O5—B1	117.29 (14)	C14—C19—H19	119.6
N2—C6—C7	112.28 (14)	C18—C19—H19	119.6
N2—C6—H6A	109.1	N2—C20—C21	111.08 (12)
C7—C6—H6A	109.1	N2—C20—H20A	109.4
N2—C6—H6B	109.1	C21—C20—H20A	109.4
C7—C6—H6B	109.1	N2—C20—H20B	109.4
H6A—C6—H6B	107.9	C21—C20—H20B	109.4
C12—C7—C8	118.56 (17)	H20A—C20—H20B	108.0
C12—C7—C6	119.24 (17)	C22—C21—C26	119.04 (17)
C8—C7—C6	122.19 (16)	C22—C21—C20	119.51 (15)
O5—C8—C9	118.10 (17)	C26—C21—C20	121.44 (15)
O5—C8—C7	121.60 (15)	C23—C22—C21	120.91 (19)
C9—C8—C7	120.28 (18)	C23—C22—H22	119.5
C10—C9—C8	119.6 (2)	C21—C22—H22	119.5
C10—C9—H9	120.2	C24—C23—C22	119.8 (2)
C8—C9—H9	120.2	C24—C23—H23	120.1
C11—C10—C9	120.7 (2)	C22—C23—H23	120.1
C11—C10—H10	119.6	C23—C24—C25	120.5 (2)
C9—C10—H10	119.6	C23—C24—H24	119.7
C12—C11—C10	119.7 (2)	C25—C24—H24	119.7
C12—C11—H11	120.1	C26—C25—C24	119.70 (19)
C10—C11—H11	120.1	C26—C25—H25	120.2
C11—C12—C7	121.0 (2)	C24—C25—H25	120.2
C11—C12—H12	119.5	O3—C26—C25	118.06 (16)
C7—C12—H12	119.5	O3—C26—C21	121.82 (16)
C14—C13—N2	115.15 (13)	C25—C26—C21	120.04 (18)
C14—C13—H13A	108.5	O4—C27—H27A	109.5
N2—C13—H13A	108.5	O4—C27—H27B	109.5
C14—C13—H13B	108.5	H27A—C27—H27B	109.5
N2—C13—H13B	108.5	O4—C27—H27C	109.5
H13A—C13—H13B	107.5	H27A—C27—H27C	109.5
C19—C14—C15	118.36 (17)	H27B—C27—H27C	109.5
C19—C14—C13	121.31 (17)		

### Hydrogen-bond geometry (Å, °)

**Table:**

*D*—H···*A*	*D*—H	H···*A*	*D*···*A*	*D*—H···*A*
C22—H22···O5^i^	0.93	2.54	3.399 (2)	153

Symmetry codes: (i) −*x*, *y*+1/2, −*z*+1/2.
